# Construction of an immune‐related LncRNA signature with prognostic significance for bladder cancer

**DOI:** 10.1111/jcmm.16494

**Published:** 2021-04-01

**Authors:** Wen‐Jie Luo, Xi Tian, Wen‐Hao Xu, Yuan‐Yuan Qu, Wen‐Kai Zhu, Jie Wu, Chun‐Guang Ma, Hai‐Liang Zhang, Ding‐Wei Ye, Yi‐Ping Zhu

**Affiliations:** ^1^ Department of Urology Fudan University Shanghai Cancer Center Shanghai China; ^2^ Department of Oncology Shanghai Medical College Fudan University Shanghai China

**Keywords:** bladder cancer, immunity, lncRNA signature, pan‐cancers, prognosis

## Abstract

Bladder cancer (BLCA) is one of the most common urological cancer with increasing cases and deaths every year. In the present study, we aim to construct an immune‐related prognostic lncRNA signature (IRPLS) in bladder cancer (BLCA) patients and explore its immunogenomic implications in pan‐cancers. First, the immune‐related differentially expressed lncRNAs (IRDELs) were identified by ‘limma’ R package and the score of IRPLS in every patient were evaluated by Cox regression. The dysregulation of IRDELs expression between cancer and para‐cancer normal tissues was validated through RT‐qPCR. Then, we further explore the biological functions of a novel lncRNA from IRPLS, RP11‐89 in BLCA using CCK8 assay, Transwell assay and Apoptosis analysis, which indicated that RP11‐89 was able to promote cell proliferation and invasive capacity while inhibits cell apoptosis in BLCA. In addition, we performed bioinformatic methods and RIP to investigate and validate the RP11‐89/miR‐27a‐3p/PPARγ pathway in order to explore the mechanism. Next, CIBERSORT and ESTIMATE algorithm were used to evaluate abundance of tumour‐infiltrating immune cells and scores of tumour environment elements in BLCA with different level of IRPLS risk scores. Finally, multiple bioinformatic methods were performed to show us the immune landscape of these four lncRNAs for pan‐cancers. In conclusion, this study first constructed an immune‐related prognostic lncRNA signature, which consists of RP11‐89, PSORS1C3, LINC02672 and MIR100HG and might shed lights on novel targets for individualized immunotherapy for BLCA patients.

## 1 INTRODUCTION

Bladder cancer (BLCA) is the most common urinary malignancy in the United States, and approximately 814,00 new cases and 17,980 deaths have been recorded in the 2020s.^1^ There are two major subtypes: non‐muscle‐invasive bladder cancer (NMIBC) and muscle‐invasive bladder cancer (MIBC). Currently, clinicians prefer to undertake transurethral resection of the bladder tumour followed by intravesical instillations of chemotherapy or immunotherapy as the therapeutic approach for NMIBC.^2^, ^3^ Radical cystectomy remains the gold standard, and the most common treatment offered for the management of primary MIBC.^4^ Recently, studies have increasingly focused on novel therapeutic and diagnostic methods for BLCA.^5^, ^6^, ^7^ However, effective potential targets and prediction models remain largely undetermined.

Long non‐coding RNAs (lncRNAs) are non‐coding RNAs ranging in length from 200 nucleotides to 100 kilobases that have diverse regulatory mechanisms in gene expression, such as chromatin modification and transcriptional and post‐transcriptional processing.^8^, ^9^ Recently, lncRNAs have been found to be associated with the tumour progression and immune microenvironment.^10^, ^11^, ^12^ For example, seminal work from Wang et al showed lnc‐DC is required for normal dendritic cell differentiation and function.^13^ Furthermore, lnc‐THRIL regulates tumour necrosis factor‐α (TNF‐α) expression through interactions with hnRNPL during innate activation of THP1 macrophages.^14^ Additionally, a great number of lncRNAs with prognostic significance have been identified,^10^, ^15^, ^16^ which emphasizes the need to identify more accurate biomarkers and establish an effective prediction model of BLCA.

Increasing evidence indicates that complex interactions are involved between cancer cells and immunity in regards to immune checkpoint genes,^17^ tumour‐infiltrating lymphocytes (TILs) ^18^ and tumour‐predicted neoantigen.^19^, ^20^ In recent years, immunotherapy has dramatically improved the treatment options for various cancers and has significantly prolonged the overall survival of treated patients. Pharmacological manipulation of the physiological immune checkpoints is one of the most promising immunotherapeutic approaches. Researchers are attempting to approve antibodies targeting checkpoint molecules such as cytotoxic T‐lymphocyte antigen 4 (CTLA4),^21^ programmed cell death 1 (PD1) ^22^ and programmed cell death ligand 1 (PD‐L1), to block major antitumour activity. However, a limited number of patients with advanced/metastatic cancer respond to ICIs,^23^ thus exposing the remaining patients to potentially ineffective, toxic and expensive treatments.^24^, ^25^ The identification of predictive factors determining the response efficiency to immunotherapy and immunogenomic landscape analysis for cancers are becoming increasingly critical. In the evolving era of immunotherapy, this work is devoted to exploring novel immune‐related lncRNAs, which have potential prognostic value for BLCA patients and might facilitate evidence‐based guidance for personalized immunotherapy. We hypothesized IRPLS as novel potential immune checkpoint targets, which may indicate therapeutic response and provide clinical strategies for the individualization of immunotherapy.

## 2 MATERIALS AND METHODS

### 2.1 Data collection and processing

We downloaded gene expression data sets of bladder urothelial carcinoma (BLCA cohort) from The Cancer Genome Atlas (TCGA) Research Network. We also obtained BLCA lncRNA expression data (GSE89006) from the Gene Expression Omnibus (GEO).^26^

### 2.2 Tissue samples and cell culture

We collected 49 bladder cancer samples and their adjacent normal tissue samples from patients with BLCA after radical resection in Fudan University Shanghai Cancer Center (FUSCC) and obtained human bladder cancer cell lines RT‐4, UM‐UC‐3, 5637, SCaBER, SW780, T24, MGH‐U3 and human immortalized normal urothelium cell line SV‐HUC‐1 from the Cell Bank of the Chinese Academy of Science (Shanghai, China) and American Type Culture Collection (Manassas, VA, USA). The T24 and RT‐4 cells were maintained in McCoy's 5 A Medium (Gibco, China). The UM‐UC‐3 and SW780 cells were maintained in MEM medium (Gibco, China). The 5637, SCaBER and MGH‐U3 cells were maintained in RPMI‐1640 medium (Gibco, China). The SV‐HUC‐1 cell was maintained in F12K medium (Gibco, China). All medium was supplemented with 1% penicillin G sodium/ streptomycin sulphate and 10% foetal bovine serum (FBS) (Gibco, Australia). All cells were grown in a humidified atmosphere consisting of 5% CO2 and 95% air at 37 ℃.

### 2.3 Quantitative reverse‐transcription polymerase chain reaction (qRT‐PCR) and analysis

Total RNA from bladder cancer cell lines and SV‐HUC‐1, BLCA tumour tissue and their adjacent normal tissue was extracted using TRIzol (Invitrogen), reverse‐transcribed to cDNA. Human LINC026672 cDNA fragments were amplified using the following primers: F, 50‐AAA CCC AGA GCC TTC CCT CAA‐30; and R, 50‐CTG TGG CTT GTG CAG CAG TGA‐30. Human RP11‐89 cDNA fragments were amplified using the following primers: F, 50‐GCT CTG GCC TGA AGC AGT ACT‐30; and R, 50‐AGA CAT TCA CAC CCC CAT ACT CTT C‐30. Human PSORS1C3 cDNA fragments were amplified using the following primers: F, 50‐GAG GTA ACT GAC GGA CGG CC‐30; and R, 50‐CTGGGGAATCTGGCAGGTTTT‐30. Human MIR100HG cDNA fragments were amplified using the following primers: F, 50‐TTT TGG AAG CGC AGA AGT TTT CTC CT‐30; and R, 50‐AGA AGC GAG GAA GCC AAG TTT ATG AG‐30. Unpaired Student's t test (normally distributed) or the Mann‐Whitney U test (non‐normally distributed) was utilized to analyse differences.

### 2.4 Identification of immune‑related differentially expressed lncRNAs

The ‘limma’ R package^27^ was used to generate the p‐value and fold change (FC) for each lncRNA between BLCA tissue and normal tissue. And we defined those with p‐value ≤ 0.05 and |log2 FC| ≥ 1 as differentially expressed lncRNAs. The single‐sample gene set enrichment analysis (ssGSEA) algorithm^28^ was carried out to evaluate the immune relevance of patients in TCGA‐BLCA cohort by quantifying the enrichment levels of the 29 immune‐associated gene sets^29^ (Table S1). And the patients’ score was provided in Table S2. The ‘limma’ R package was used again to obtain immune‐related lncRNAs (p‐value ≤ 0.05 and |log2 FC| ≥ 1) between high and low score groups. The overlapping lncRNAs among different groups were determined via Venn diagrams.

### 2.5 Construction of immune‐related lncRNA signature

We performed univariable Cox proportional hazards regression model analyses for overall survival (OS) and recurrence‐free survival (RFS) to obtain prognosis‐related lncRNAs. Multivariable Cox proportional hazards regression model analyses were used^30^ to build immune‐related lncRNA signature (IRPLS) and determine the best cut‐off value to distinguish BLCA patients into high‐risk group and low‐risk group. Kaplan‐Meier (KM) method and log‐rank tests were used to calculate differences between groups. The receiver operating characteristic curve (ROC) was constructed to validate the predictive ability of the IRPLS using ‘survival ROC’ R package.

### 2.6 Cell counting kit (CCK)‐8 assay

The cell proliferation ability was tested by Cell Counting Kit‐8 (Dojindo, CK04). First, we seeded cells into 96‐well plates (5000 cells/well) with complete growth medium. After incubation for 24, 48, 72 and 96 h, respectively, 10 mL CCK‐8 was added into each well and then, the cells were cultured for an additional 2 h. Finally, the absorbance of the samples was measured at 450 nm using Microplate Spectrophotometer (BioTek, VT, USA).

### 2.7 Transwell assay

Cell invasive capacity was determined using Transwell chambers (BD Biosciences). A total of 20 000 cells were plated in the top of a polycarbonate Transwell filter with 200 mL medium without foetal bovine serum. We fill the lower compartment with 500 mL culture medium with foetal bovine serum. After incubation for 24 hours (for 5637 cells) and 36 hours (for T24 cells), we stained the migrated cells using crystal violet and counted using Imagine J.

### 2.8 Cell apoptosis assay

Cell apoptosis was evaluated by an Annexin V‐APC/7‐AAD kit. Flow cytometry was used to examine cells harvested after incubation with Annexin V‐FITC/PI double staining.

### 2.9 RNA immunoprecipitation (RIP) assay

We validated the relationship between lncRNA RP11‐89 and miR‐27A‐3p via a Magna RIP RNA‐Binding Protein Immunoprecipitation Kit (Millipore, USA). Anti‐AGO2 and control IgG (Millipore, USA) were utilized for the RIP assay, and we evaluate the coprecipitated RNAs via cDNA synthesis and qRT‐PCR.

### 2.10 Immune‐related analysis in bladder cancer

CIBERSORT algorithm in R software was utilized to estimate the abundance of tumour‐infiltrating immune cells.^31^ And we calculated immune scores and stromal scores for each sample applying the ‘Estimation of STromal and Immune cells in MAlignant Tumours using Expression data’ (ESTIMATE) algorithm.^18^

### 2.11 Gene set enrichment analyses

We performed the R category (version 2.10.1) package to integrate the TCGA‐BLCA data sets with gene set enrichment analysis (GSEA)^32^ or functional enrichment analysis. GSEA was utilized to determine whether the set of genes was at the top of the sorted table or at the bottom when enriched. In order to optimize the results, GSEA detected changes in the expression of a gene set rather than a single gene and contained subtle expression changes, using adjusted p‐values and the false discovery rate (FDR) meth. Finally, we obtained statistically significant involved hub genes via an adjusted p‐value < 0.01 and FDR < 0.25. R software (Version 3.3.2) were conducted to finish statistical analysis and graphical plotting.

### 2.12 Immunogenomic landscape analysis in pan‐cancers

The ssGSEA analysis was utilized to evaluate expression of checkpoint genes in pan‐cancer of TCGA cohort according to 29 immune‐associated gene sets^29^ (Table S1). Abundance of 28 kinds of tumour‐infiltrating lymphocytes (TILs) and predicted SNV (single‐nucleotide variants)‐derived Neoantigen of pan‐cancer in TCGA cohorts were obtained from ‘https://tcia.at/home’ and ‘https://gdc. cancer. gov/about‐data/publications/panimmune’. Pearson correlation tests were performed to evaluate association of expression between lncRNAs and checkpoint genes, tumour‐infiltrating lymphocytes (TILs) and tumour‐predicted SNV neoantigen.

## 3 RESULTS

### 3.1 Identification of immune‑related differentially expressed lncRNAs

**FIGURE 1:**
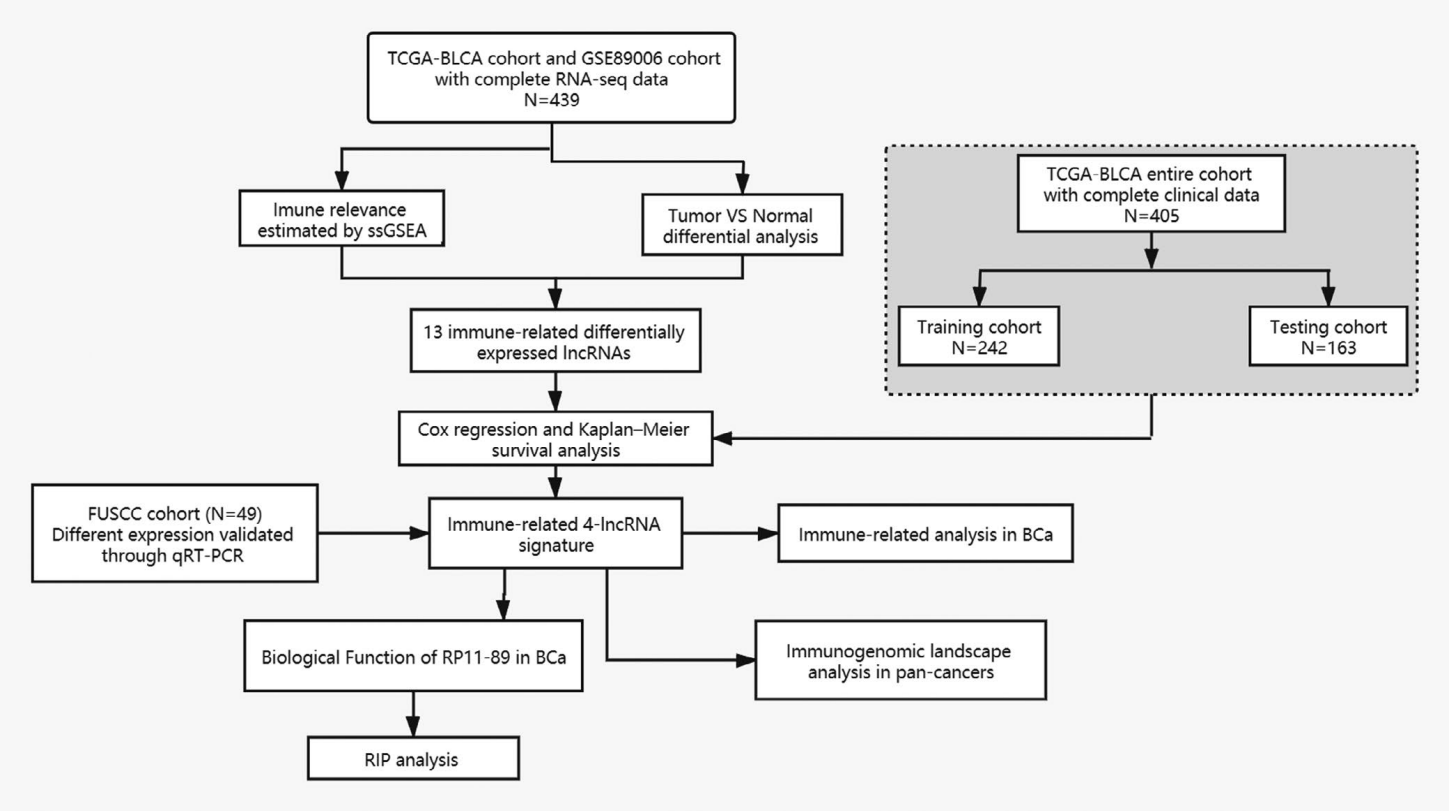
Flow chart of this study

**FIGURE 2:**
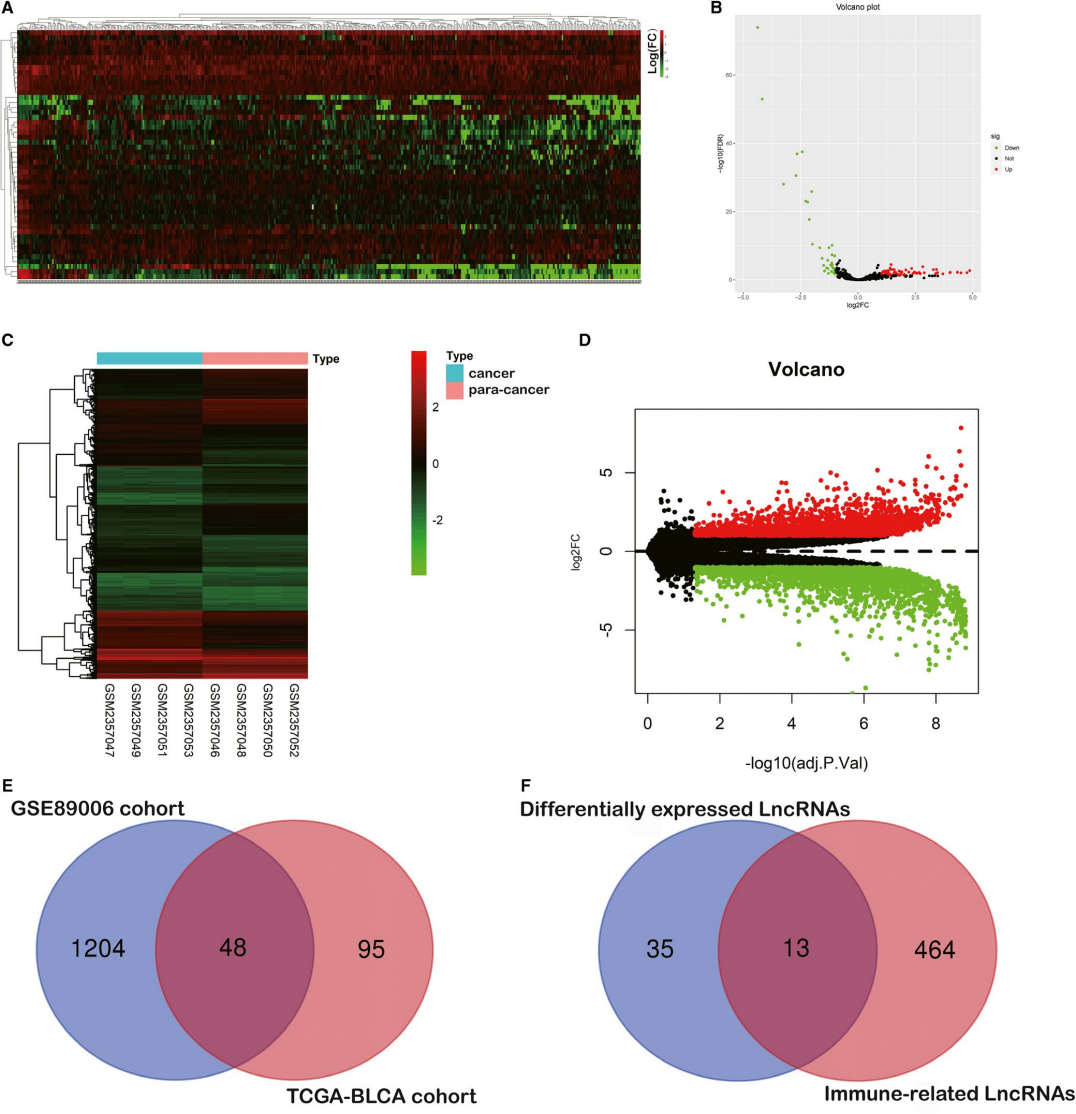
Identification of immune‐related differentially expressed lncRNAs (ICDELs). A and B, The genomic heat map and volcano plot was presented to show the 143 differentially expressed lncRNAs identified from the TCGA cohort. C and D, The genomic heat map and volcano plot were presented to show 1,252 differentially expressed lncRNAs identified from the GSE89006 cohort. E and F, A total of 48 candidate lncRNAs were overlapped between the two major cohorts, and 13 candidates were identified as the immune‐related differentially expressed lncRNAs

Figure 1 was depicted to demonstrate the overall design for this study. As shown in Figure 2A‐D, A total of 416 BLCA patients and 23 healthy patients from both the TCGA‐BLCA and GSE89006 cohorts were included in this study. Then, 16,481 lncRNAs (from the TCGA cohort) and 10,863 lncRNAs (from the GSE89006 cohort) were identified by mapping the ENSEMBL ID with references to the Gencode database (https://www.gencodegenes.org/human/). As Figure 2E shows, 1,347 differentially expressed lncRNAs (DELs) between BLCA tissue and normal tissue were identified, and 48 candidate lncRNAs overlapped between the two major cohorts. Furthermore, 477 lncRNAs were recognized as immune‐related lncRNAs by ssGSEA (Figure 2F). Finally, 13 candidates were identified for further research.

### 3.2 Construction of IRPLS and validation of lncRNAs dysregulation in BLCA

**FIGURE 3:**
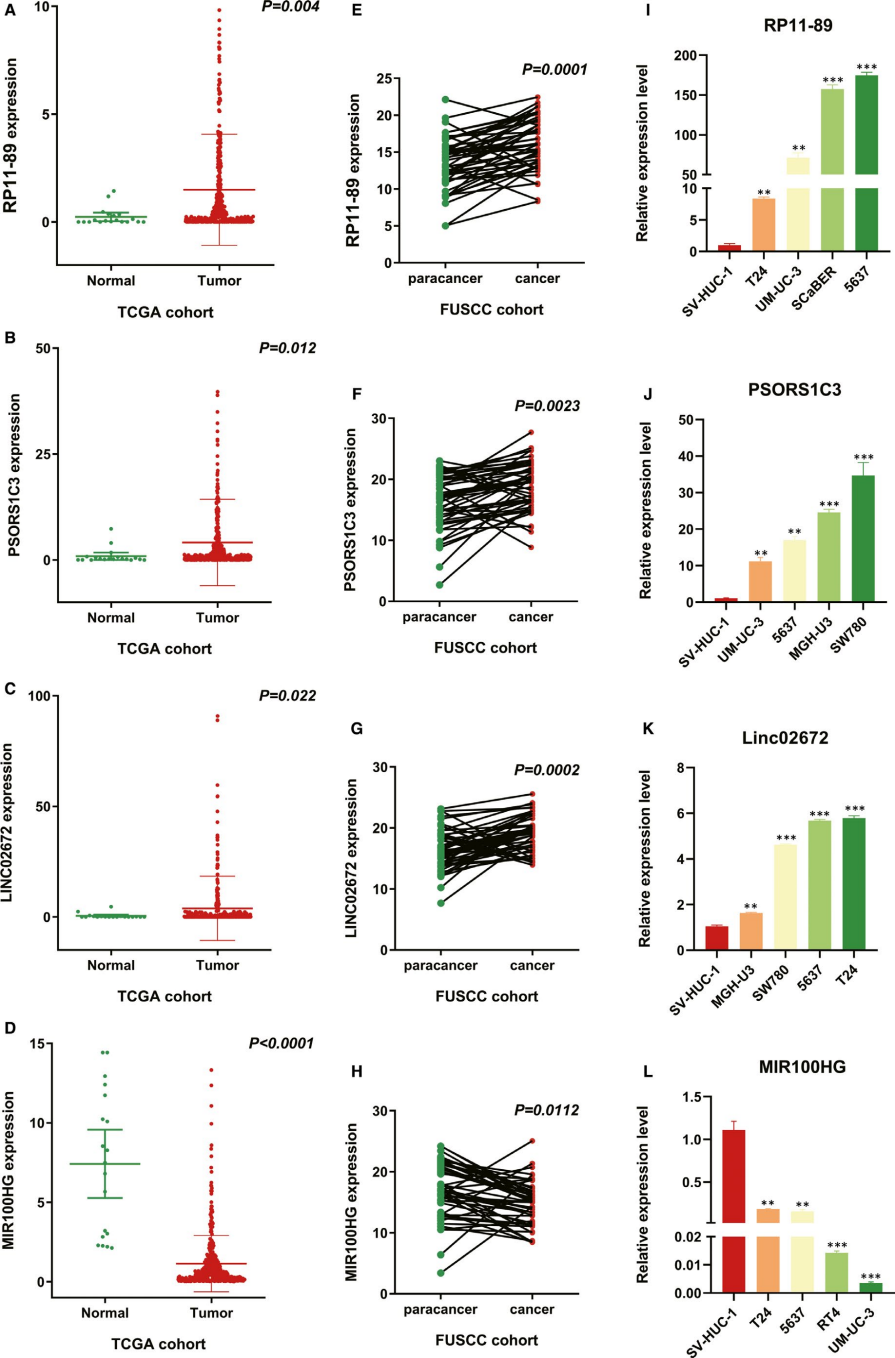
Different expression of RP11‐89, PSORS1C3, MIR100HG, and LINC02672 in BLCA. A‐H, RT‐qPCR was performed to show the expression level of RP11‐89, PSORS1C3, LINC02672 and MIR100HG in tumour samples compared with normal samples (from the TCGA cohort) and in cancer tissues compared with paired para‐cancerous tissues (from the FUSCC cohort). I‐L, The RT‐qPCR results were used to demonstrate the expression level of RP11‐89, PSORS1C3, LINC02672 and MIR100HG in different BLCA cell lines. *P <.05, **P <.01, ***P <.001

As shown in Table 1, we conducted multivariate Cox regression to identify four significant lncRNAs from 13 hub lncRNAs. The IRPLS was constructed by these four lncRNAs containing RP11‐89, PSORS1C3, LINC02672 and MIR100HG. All patients in the TCGA cohort were stratified into low‐risk or high‐risk groups using a median IRPLS risk scores, which was calculated as follows: riskScore = expression level of RP11‐89*(−0.0955) + expression level of PSORS1C3*(−0.0319) + expression level of LINC02672*(−0.0127) + expression level of MIR100HG*0. 0577. And the patient characteristics in both TCGA and FUSCC cohort were summarized in Table 2. Figure 3A‐D showed us the comparison of lncRNAs expressions between normal samples and tumour samples in TCGA cohort. RP11‐89, PSORS1C3 and LINC02672 were highly expressed in tumour samples while MIR100HG was highly expressed in normal samples. As shown in Figure 3E–L, we measured the relative expression of four lncRNAs of IRPLS in 49 pairs of specimens from the FUSCC cohort and cell lines including RT‐4, UM‐UC‐3, 5637, SCaBER, SW780, T24, MGH‐U3 and SV‐HUC‐1 cells. The RT‐qPCR results demonstrated that RP11‐89, PSORS1C3 and LINC02672 were significantly up‐regulated in RT‐4, UM‐UC‐3, 5637 and SCaBER cell lines compared with SV‐HUC‐1 cells and in cancer tissues compared with paired para‐cancerous tissues (*P* <.05). Conversely, MIR100HG was significantly down‐regulated in RT‐4, UM‐UC‐3, T24 and 5637 cell lines compared with SV‐HUC‐1 cells and in cancer tissues compared with paired para‐cancerous tissues (*P* <.05).

**TABLE 1:** Survival analysis and immune relevance of IRLPS

LncRNA	HR	*P*‐value (uniCox)	*P*‐value (multiCox)	*P*‐value (KM)	*P*‐value (ssGSEA)
RP11‐89	0.79625	0.010613	0.042812	0.0451	<0.0001
PSORS1C3	0.828144	0.003685	0.056288	0.0155	0.001306
LINC02672	0.861944	0.021461	0.218217	0.6095	<0.0001
MIR100HG	1.28655	0.002121	0.077125	0.0007	<0.0001

**TABLE 2:** Clinicopathological characteristics of BLCA patients in the discovery cohort. (TCGA cohort and Fudan University Shanghai Cancer Center cohort)

Characteristics	FUSCC cohort (N = 49)	TCGA cohort	
		Training cohort (N = 246)	Testing cohort (N = 163)
N (%)			
Age			
<70 years	30(61.2)	126(51.2)	91(55.8)
≥70 years	19(38.8)	120(48.8)	72(44.2)
Gender			
Male	35 (71.4)	178(72.3)	124(76.1)
Female	14 (28.6)	68(27.7)	39(23.9)
Tumour stage			
I‐II	26(56.5)	74(30.3)	57(35.0)
III‐IV	20(43.5)	170(69.7)	106(65.0)
T stage^a^			
T1 ‐ T2	25(54.3)	68(30.1)	53(35.6)
T3 ‐ T4	21(45.7)	158(69.9)	96(64.4)
N stage^a^			
N0‐1	36(78.2)	169(78.2)	113(75.3)
N2‐3	10(21.8)	47(21.8)	37(24.7)
M stage^a^			
M0	35(77.7)	111(93.3)	88(96.7)
M1	10(22.3)	8(6.7)	3(3.2)
Grade			
Low	14(31.8)	13(5.4)	7(4.3)
High	30(68.2)	231(94.6)	155(95.7)
Subtype			
Papillary	7(16.7)	78(31.7)	53(33.5)
Non‐Papillary	35(83.3)	168(68.3)	105(66.5)

^a^ TNM scoring system: Tumour size, lymph nodes affected, metastases.

### 3.3 Confirmation of IRPLS as an independent predictor and establishment of a novel nomogram for predicting OS in BLCA

**FIGURE 4:**
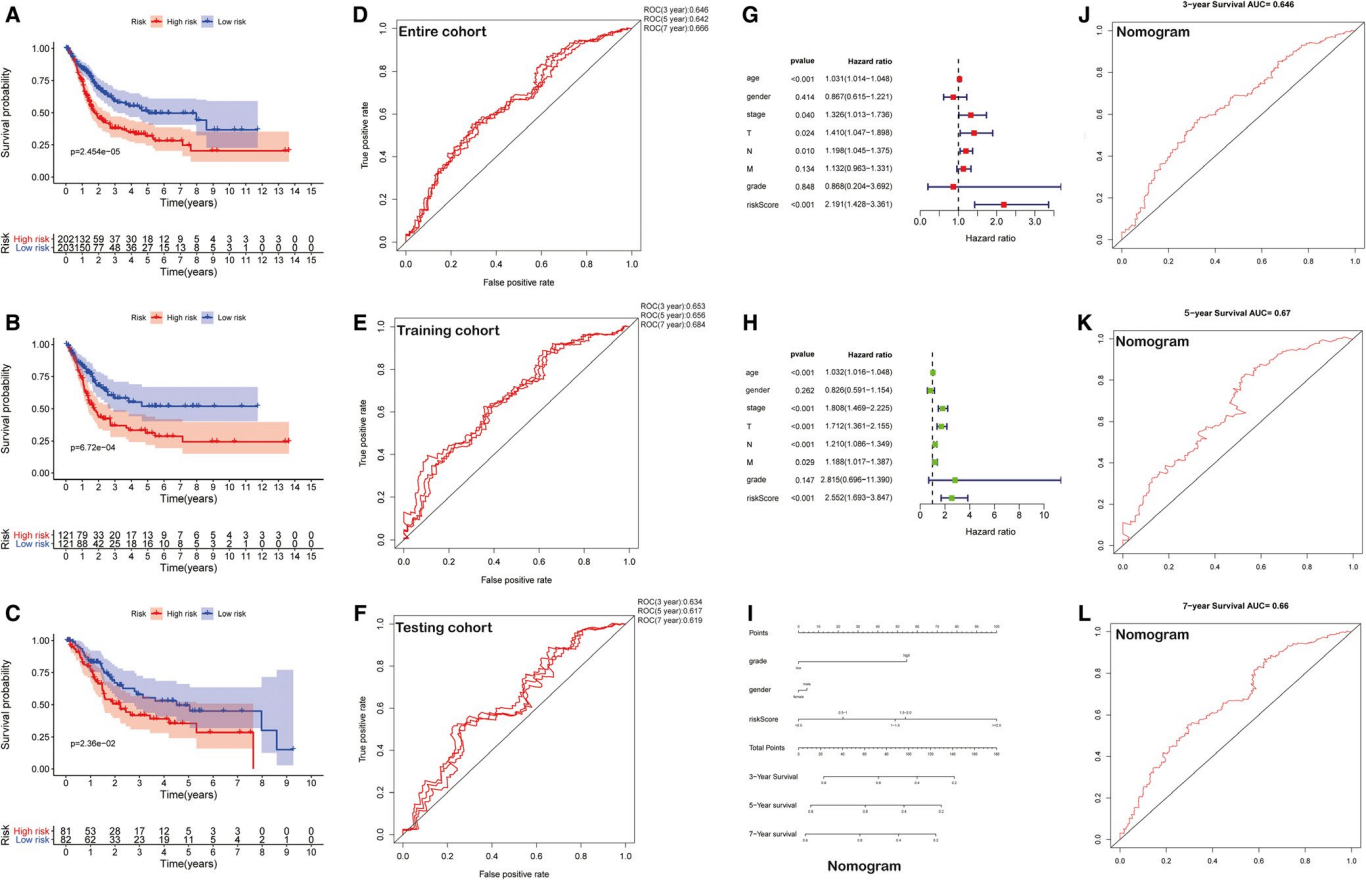
Validation of IRPLS as an independent predictor and construction of a novel nomogram for predicting OS. A‐C, Kaplan‐Meier survival analysis was used to show the significant prognostic value in the entire cohort, the testing cohort and the training cohort. D‐F, ROC analysis showed the AUC values for 3‐, 5‐ and 7‐year OS of 3 cohorts. G‐H, Both the univariate Cox regression analysis and the multivariate Cox regression analysis integrating major clinical characteristics with IRPLS were performed to demonstrate the independence factors for OS in BLCA. I, A nomogram was developed to predict 3‐, 5‐ and 7‐year OS using the IRPLS riskScore and the aforementioned clinical factors. J‐L, ROC analysis showed the AUC values for 3‐, 5‐ and 7‐year OS of the nomogram

The entire TCGA‐BLCA cohort was randomly divided into training and testing cohorts at a cut‐off value of 3:2. In the training cohort (N = 242), KM survival curves indicated that the low‐risk patients lived longer than the high‐risk patients (Figure 4B) (*P* <.001). Time‐dependent ROC analysis showed an appropriate accuracy of IRPLS in predicting OS, and the area under the ROC curve (AUC) was 0.653 at 3 years, 0.656 at 5 years and 0. 684 at 7 years (Figure 4E). For further validation, we confirmed that the results in the training cohort were consistent with the outcomes in the testing cohort and in the entire cohort, indicating that the high‐risk patients were associated with poorer prognosis. In the testing cohort (N = 163), the significant prognostic value was *P* =.0236 (Figure 4C) and AUC values for 3‐, 5‐ and 7‐year OS were 0.634, 0.617 and 0.619, respectively (Figure 4F). In the entire cohort (N = 405), the significant prognostic value was *P* <.001 (Figure 4A) and AUC values for 3‐, 5‐ and 7‐year OS were 0.646, 0.642 and 0.666, respectively (Figure 4D). By integrating major clinical characteristics with IRPLS, both the univariate Cox regression analysis and the multivariate Cox regression analysis demonstrated that sex and tumour grade were also responsible for OS in BLCA (Figure 4G‐H) (*P* <.05). Furthermore, we developed a nomogram to predict 3‐, 5‐ and 7‐year OS using the IRPLS risk Score and the aforementioned clinical factors (Figure 4I). The AUC values for 3‐, 5‐ and 7‐year OS in the nomogram were 0.646, 0.67 and 0.66, respectively.

### 3.4 RP11‐89 promotes BLCA progression via increasing cell proliferation and invasive capacity while suppressing cell apoptosis

**FIGURE 5:**
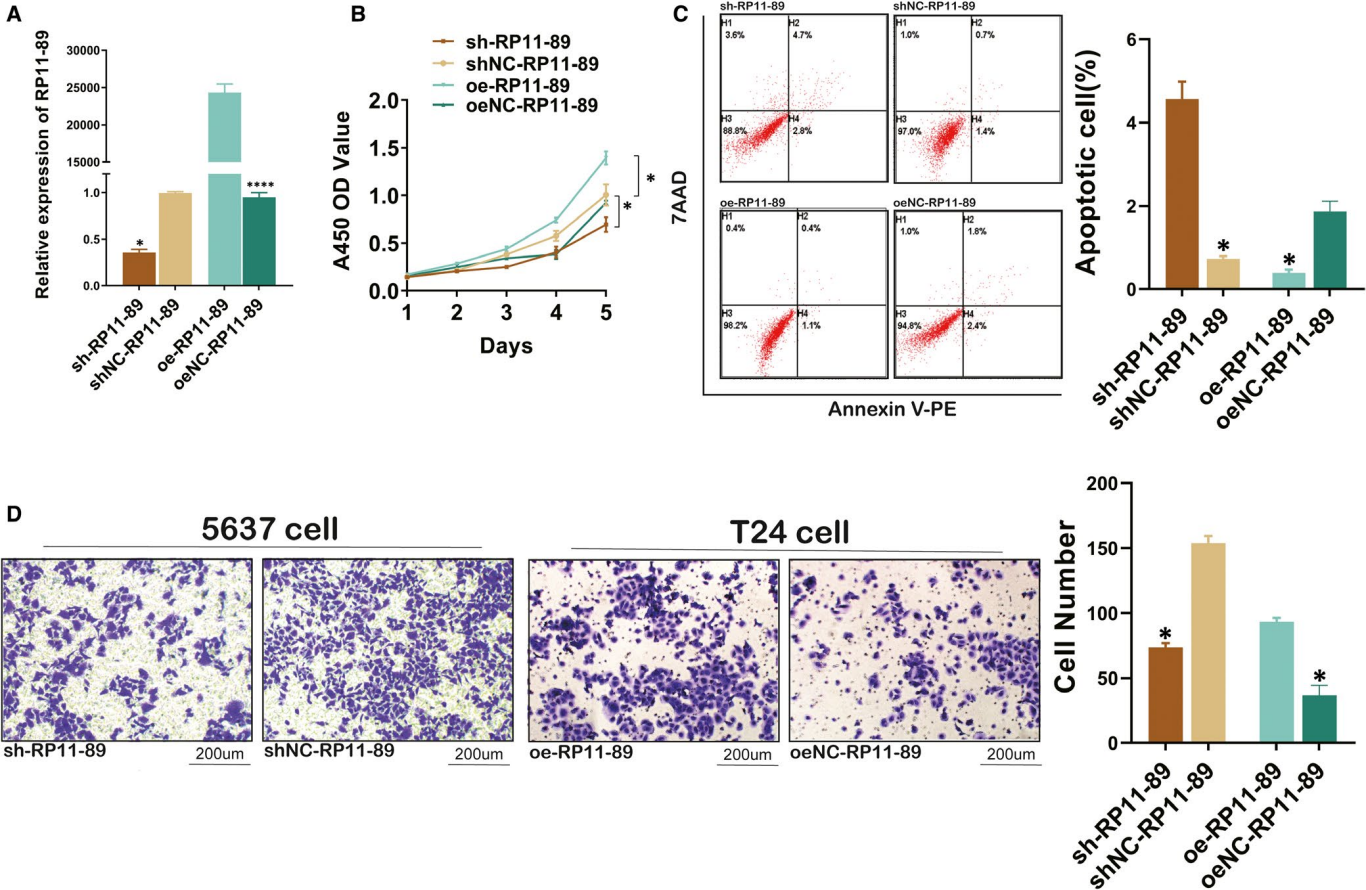
RP11‐89 promotes BLCA progression. A, RT‐qPCR was conducted to verify the transfection efficiency. B, CCK8 assay exhibited the cell viability of 5637 cells treated with sh‐RP11‐89 and T24 cells treated with overexpression plasmid compared with respective control. C, Apoptosis analysis was performed to determine the cell apoptosis of cells transduced with different plasmid. D, Transwell assay revealed different cell invasive capacity when RP11‐89 was knocked down or over‐expressed in BLCA cell lines. All data are expressed as mean SD of three independent experiments; *P <.05, **P <.01, ***P <.001, ****P <.0001

We found one of IRPLS lncRNAs, RP11‐89 was rarely investigated in BLCA or other tumours and evaluated its biological function in BLCA. Furthermore, we transfected 5637 cell with RP11‐89 shRNA plasmid and T24 cell with overexpression plasmid, and we use RT‐qPCR to verify transfection efficiency (Figure 5A). As the result of CCK8 assay presented, 5637 cells treated with sh‐RP11‐89 exhibited attenuated cell viability when compared with 5637 cells treated with sh‐RP11‐89 NC and T24 cells treated with overexpression plasmid exhibited increased cell viability when compared with T24 cells treated with NC plasmid (Figure 5B) (*P* <.05). Furthermore, we performed apoptosis analysis, which showed that RP11‐89 depletion in 5637 cells induce cell early apoptosis (Figure 5C), while RP11‐89 enrichment in T24 cells inhibited cell early apoptosis (*P* <.05). Transwell assay revealed that knock‐down of RP11‐89 significantly suppressed cell invasive capacity and overexpression significantly promoted cell invasive capacity compared with the respective controls. (Figure 5D) (*P* <.05). Taken together, the results indicated that RP11‐89 is an oncogenic lncRNA, which promotes cell proliferation and invasive capacity while inhibits cell cycle apoptosis in BLCA.

### 3.5 RP11‐89 targeted miR‐27a‐3p and up‐regulate PPARγ expression in BLCA

**FIGURE 6:**
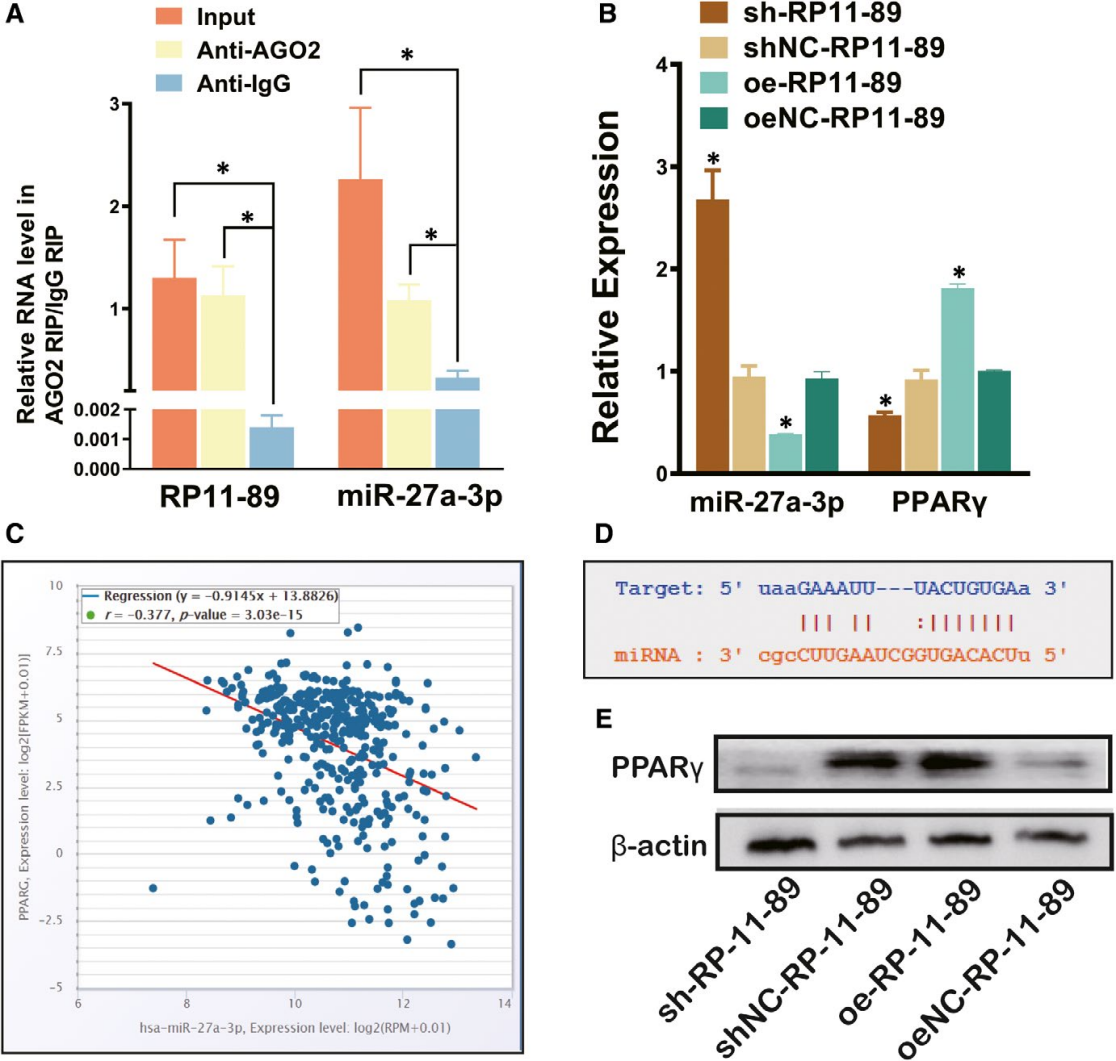
RP11‐89 targeted miR‐27a‐3p and up‐regulate PPARγ expression in BLCA. A,RIP analysis was used to determine the relationship between RP11‐89 and miR‐27a‐3p. B and E, RT‐qPCR and Western blot analysis were performed to evaluate the expression level of miR‐27a‐3p and PPARγ in cells dealed with sh‐RP11‐89 or overexpression plasmid and the respective control. C, TCGA‐BLCA analysis shows the negative regulation of expression level between miR‐27a‐3p and PPARγ in BLCA. D, Bioinformatic analysis in starBase database shows the binding sites between miR‐27a‐3p and 3’UTR of PPARγ. All data are expressed as mean SD of three independent experiments; *P <.05, **P <.01, ***P <.001

Through the miRcode database (Table 3), we predicted downstream miRNAs of RP‐1189 and identified miR‐129‐5p as a potential target of RP‐1189. RT‐qPCR result and Western blot analysis showed that 5637 cells transduced with sh‐RP‐1189 plasmid exhibited increased miR‐27a‐3p expression but decreased PPARγ when compared to the cells treated with sh‐RP‐1189 control plasmid (Figure 6B, E) (*P* <.05). The same trends were also observed in T24 cell transfected with overexpression control plasmid in contrast to T24 cell treated with overexpression plasmid (*P* <.05). In order to validate the relationship between miR‐27a‐3p and RP11‐89, we performed the AGO2 immunoprecipitation assay, which showed that the AGO2 antibody was able to pull down both endogenous miR‐27a‐3p and RP11‐89 (Figure 6A). We performed bioinformatic method to analysis targeted genes of miR‐27a‐3p in starBase database (http://starbase.sysu.edu.cn/), which showed that miR‐27a‐3p was able to bind with PPARγ in 3′‐UTR region and caused the negative regulation of PPARγ (Figure 6D). It could be concluded from the findings mentioned above that RP11‐89 might ‘sponge’ miR‐27a‐3p and up‐regulate PPARγ expression in BLCA.

**TABLE 3:** Prediction of downstream miRNA miR‐27a/3p regulated by RP11‐89 in miRcode database

microRNA family	Seed position	Seed type	Transcript region	Repeat	Conservation		
					Primates	Mammals	Othervert.
miR‐27abc/27a‐3p	chr2:45 148 866	7‐mer‐A1	ncRNA	yes	56%	0%	0%
miR‐27abc/27a‐3p	chr2:45 149 818	8‐mer	ncRNA	no	78%	74%	23%
miR‐27abc/27a‐3p	chr2:45 157 174	7‐mer‐A1	ncRNA	no	89%	78%	31%

Abbreviations: miR, microRNA; ncRNA, non‐coding RNA.

### 3.6 Immune relationship between IRPLS and BLCA

**FIGURE 7:**
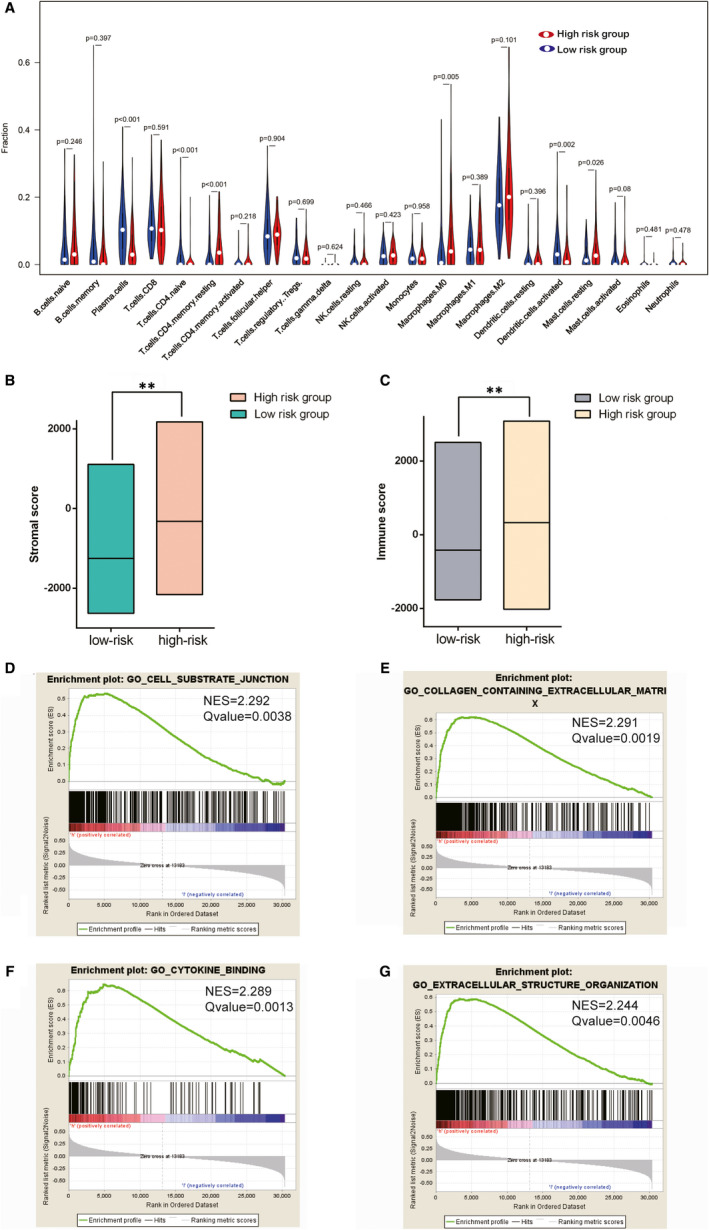
Tumour immune microenvironment and immune‐related pathways were significantly different between the high‐risk group and low‐risk group. A, Violin plots show the distribution of immune cells between the high‐risk group and low‐risk groups. B and C, Different stroma scores and immune scores were indicated between the high‐risk group and low‐risk groups. D‐G, GSEA analysis indicated the functional changes between the high‐risk group and low‐risk group. *P <.05, **P <.01, ***P <.001

As shown in Figure 7A, the distribution of immune cells was significantly different between the high‐ risk and low‐risk groups. The fraction of T cells in low‐risk group was remarkably higher than high‐risk group (*P* <.001). According to the ESTIMATE algorithm (Figure 7B‐C), the high‐risk group showed higher stroma scores and higher immune scores compared with the low‐risk group (*P* <.01). GSEA analysis indicated (Figure 7D‐G) that the functional changes between the high‐risk group and low‐risk group were mostly enriched in cell‐substrate junctions, collagen‐containing extracellular matrix, cytokine binding and extracellular structure organization (*P* <.01), which were consistent with the biological function of RP11‐89 in BLCA.

### 3.7 Immune landscape analysis of IRPLS in pan‐cancers

**FIGURE 8:**
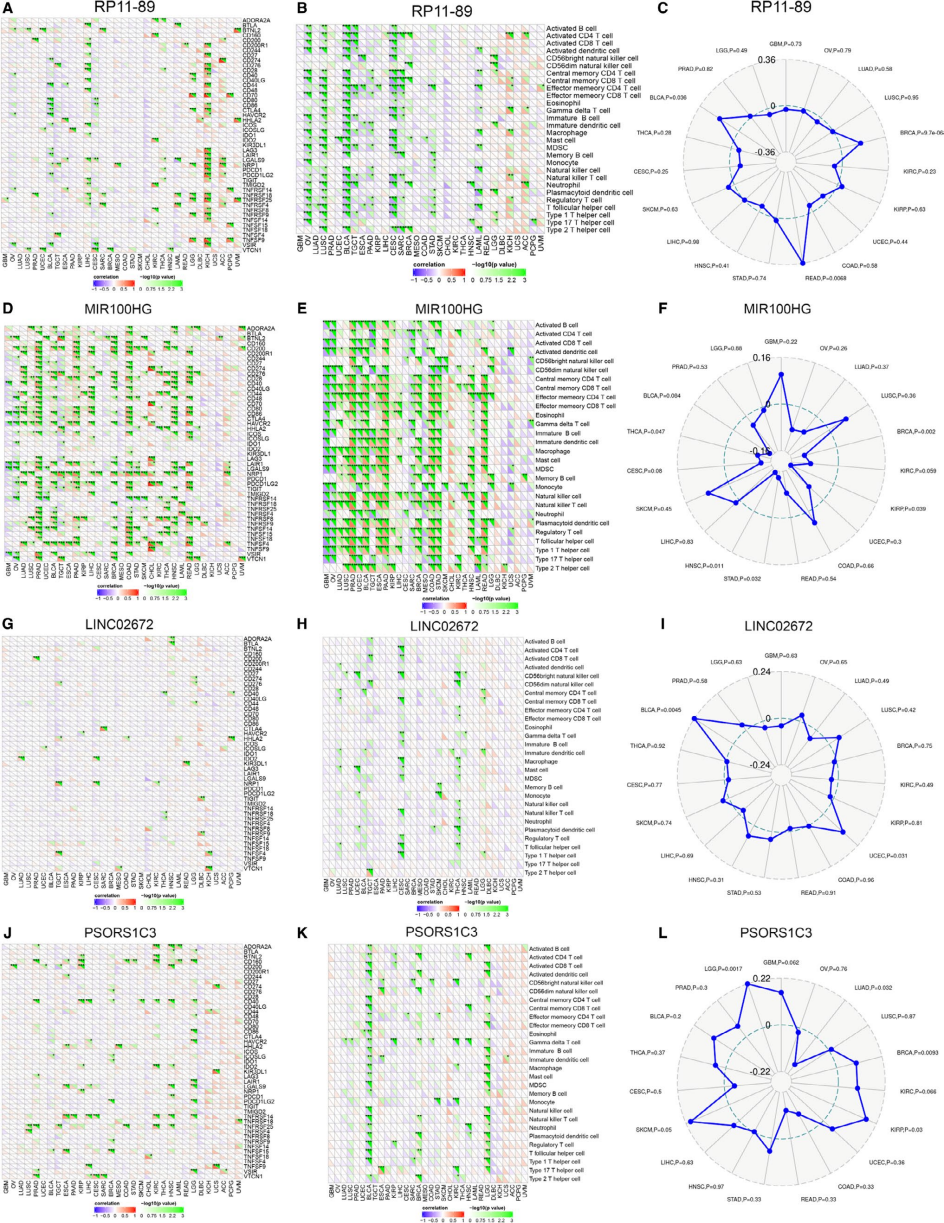
Pan‐cancer immunogenomic landscape analysis of four lncRNAs. A, D, G, J, Association between 4 lncRNAs in IRPLS and the expression level of different immune‐related genes. B, E, H, K, Association between 4 lncRNAs in IRPLS and the abundance of immune cells C, F, I, L, Association between 4 lncRNAs in IRPLS and the predicted SNV‐derived neoantigens in pan‐cancers. *P <.05, **P <.01, ***P <.001

RP11‐89 was significantly correlated with the expression of CD44, CD70 and LAGLS9 in pan‐cancers (Figure 8A) and was significantly associated with the abundance of activated CD4 T cells, activated CD8 T cells and effector memory CD4 T cells in pan‐cancers (Figure 8B). RP11‐89 was also significantly correlated with predicted SNV‐derived neoantigens (Figure 8C) of BLCA (*P* <.05) and rectal adenocarcinoma (*P* <.01). MIR100HG was significantly correlated with the expression of CD200, CD274 and CD276 in pan‐cancers (Figure 8D) and was significantly associated with the abundance of central memory CD8 T cells, effector memory CD4 T cells and effector memory CD8 T cells in pan‐cancers (Figure 8E). Furthermore, MIR100HG was significantly correlated with predicted SNV‐derived neoantigens (Figure 8F) of head and neck squamous cell carcinoma (*P* <.05), thyroid carcinoma (*P* <.05), stomach adenocarcinoma and breast cancer (*P* <.05). LINC02672 was significantly correlated with the expression of IDO1, NRP1 and TNFSF4 in pan‐cancers (Figure 6G), and it was significantly associated with the abundance of CD56 bright natural killer cells, mast cells, and immature dendritic cells in pan‐cancers (Figure 8H). And LINC02672 was also significantly correlated with predicted SNV‐derived neoantigens (Figure 8I) of bladder cancer (*P* <.01) and uterine corpus endometrial carcinoma (*P* <.05) PSORS1C3 was significantly correlated with the expression of CD160, CD40 and TNFRSF14 in pan‐cancers (Figure 8J), and it was significantly associated with the abundance of activated CD4 cells and gamma delta T cells in pan‐cancers (Figure 8K). Moreover, PSORS1C3 was significantly correlated with predicted SNV‐derived neoantigens (Figure 8L) of lower‐grade glioma (*P* <.01), uterine corpus endometrial carcinoma (*P* <.05), lung adenocarcinoma (*P* <.05), breast cancer (*P* <.01) and kidney renal papillary cell carcinoma (*P* <.05).

## 4 DISCUSSION

BLCA is one of the most common urological cancers and is a major therapeutic challenge. Identification of the molecular mechanisms related to bladder carcinogenesis is becoming crucial for novel pharmacotherapies and biomarkers for this disease. Recently, lncRNAs, originally thought to be noise from RNA transcription, have been used as biomarkers for the diagnosis, prognosis and targeted therapy of multiple cancers.^33^ In our research, we examined differentially expressed lncRNAs in multiple cohorts and screened out immune checkpoint‐related lncRNAs for the construction of a signature. Then, we performed RT‐qPCR to validate the dysregulation of immune‐related lncRNA signature (IRPLS) in BLCA. Surprisingly, Kaplan‐Meier (KM) survival analyses and time‐dependent receiver operating characteristic (ROC) curves from the training cohort, the testing cohort and the entire cohort suggested that IRPLS has good reproducibility and robustness in prognosis prediction for BLCA patients. We constructed a nomogram integrating patients' risk Scores and prognostic clinical features including grade and sex for predicting patient outcomes. The ROC curve for predicted nomogram was also calculated in order to confirm the accuracy and precision of our evaluation. Furthermore, we were attempting to investigate one of the IRPLS lncRNAs and RP11‐89 for its novelty in BLCA. Till date, few studies have investigated the potential role of RP11‐89 in cancers, one study by Chen et al, identified a Wnt pathway‐related lncRNA profile which includes RP11‐89 to be associated with the development of lung cancer.^34^ In our work, RP11‐89 was evaluated through multiple assays and the results revealed that it could promote cell proliferation and invasive capacity while inhibits cell apoptosis in BLCA via miR‐27a‐3p/PPARγ. An increasing number of studies have revealed that PPARγ, one of peroxisome proliferator‐activated receptors, plays essential role in cancer metabolism.^35^ Subsequently, more evidence has indicated PPARγ activation as a potential tumorigenic trigger in BLCA.^36^, ^37^ Otherwise, for its crucial role in cellular energy homeostasis, immune cells also require PPARγ activation to meet energy demands and regulate lipid metabolism and cell fate, which is involved in tumour immune microenvironment.^38^, ^39^

The heterogeneity of immune status in BLCA is particularly high. We evaluated the type and number of infiltrating immune cells between the high‐risk and low‐risk groups. To explore the underlying mechanisms, GSEA results showed that genes related to cell‐substrate junctions and cytokine activity were enriched in the high‐risk group. In recent years, great effort has been made in the implementation of immunotherapeutic approaches for treating various cancer types. For the first time, we characterized aspects of the tumour immune landscape related to IRPLS in pan‐cancers, including immune checkpoints, TILs and predicted SNV‐derived neoantigens, all of which can inform immunotherapy decisions. Accumulating data suggest that neoantigens are relevant targets for personalized anti‐cancer therapies.^40^, ^41^ Given the analysis of our findings, IRPLS, if validated, not only has roles in predicting the survival outcome and immune status of BLCA patients but is also tightly correlated with pan‐cancer immunity. Interestingly, the high relevance of MIR100HG with immunotherapy, which is quite ‘hot’ in the presented heat map, has been confirmed in multiple cancers including osteosarcoma, laryngeal squamous cell carcinoma, triple‐negative breast cancer and gastric cancer.^42^, ^43^, ^44^, ^45^ Furthermore, for the first time we reveal the commonality and heterogeneity of tumour immunity through immunogenomic landscape analysis of the lncRNA signature, which has the potential to contribute to the implementation of immunotherapy approaches for patients, especially those diagnosed with multiple primary cancers.

In conclusion, this study first constructed an immune‐related prognostic lncRNA signature (IRPLS), which consists of RP11‐89, PSORS1C3, LINC02672 and MIR100HG. As an oncogenic lncRNA, RP11‐89 is able to promote cell proliferation and invasive capacity while inhibit cell apoptosis in BLCA. IRPLS significantly predicts poor clinical outcomes for bladder cancer patients and immune microenvironment disorders in pan‐cancers, which might shed lights on novel targets for individualized immunotherapy.

## CONFLICT OF INTERESTS

The authors declare no competing interests.

## AUTHOR CONTRIBUTION

**Wen‐Jie Luo:** Data curation (equal); Formal analysis (equal); Writing‐original draft (equal); Writing‐review & editing (equal). **Xi Tian:** Data curation (equal); Formal analysis (equal). **Wen‐Hao Xu:** Data curation (equal); Formal analysis (equal). **Yuan‐Yuan Qu:** Investigation (equal); Methodology (equal). **Wen‐Kai Zhu:** Investigation (equal); Methodology (equal). **Jie Wu:** Software (equal). **chun‐guang Ma:** Investigation (equal). **Hai‐liang Zhang:** Funding acquisition (equal). **Ding‐Wei Ye:** Funding acquisition (equal). **Yi‐Ping Zhu:** Funding acquisition (equal).

## ETHICAL APPROVAL

The Ethics approval and consent to participate of the current study were approved and consented by the ethics committee of Fudan University Shanghai Cancer Center.

## Supporting information

Table S1

Table S2

## Data Availability

The data sets during and/or analysed during the current study available from the corresponding author on reasonable request.
