# Supplementary material for: Construction of an immune‐related LncRNA signature with prognostic significance for bladder cancer
Source: J Cell Mol Med. 2021 Apr 1;25(9):4326–39. doi: 10.1111/jcmm.16494 (PMC8093983; doi:10.1111/jcmm.16494)
Supplement: Supplementary file 2 — Table S2 [file JCMM-25-4326-s001.docx]

Supplementary table 2. Patients’ score of immune-relative genes expression estimated by ssGSEA.(based on the Check-point gene set from supplementary table 1)

| Patient id | Immune Score |
| --- | --- |
| TCGA-ZF-A9R5-01A-12R-A42T-07 | 0.339278 |
| TCGA-E7-A7PW-01A-11R-A352-07 | 0.237119 |
| TCGA-4Z-AA7N-01A-11R-A39I-07 | 0.910671 |
| TCGA-DK-A2I1-01A-11R-A180-07 | 0.594617 |
| TCGA-GC-A3RC-01A-11R-A22U-07 | 0.666129 |
| TCGA-ZF-AA52-01A-12R-A39I-07 | 0.506404 |
| TCGA-E7-A541-01A-11R-A26T-07 | 0.628023 |
| TCGA-GV-A3QK-01B-11R-A23N-07 | 0.582591 |
| TCGA-CF-A47W-01A-11R-A23W-07 | 0.195384 |
| TCGA-ZF-A9RL-01A-11R-A38B-07 | 0.153551 |
| TCGA-UY-A9PA-01A-11R-A38B-07 | 0.54738 |
| TCGA-ZF-AA4R-01A-11R-A38B-07 | 0.442175 |
| TCGA-YF-AA3M-01A-11R-A42T-07 | 0.334492 |
| TCGA-E7-A7DV-01A-11R-A33J-07 | 0.712211 |
| TCGA-DK-AA76-01A-11R-A39I-07 | 0.086625 |
| TCGA-ZF-AA4N-01A-11R-A38B-07 | 0.53733 |
| TCGA-CF-A47V-01A-11R-A23W-07 | 0.397425 |
| TCGA-K4-A4AC-01A-21R-A26T-07 | 0.527257 |
| TCGA-CU-A0YO-01A-11R-A10U-07 | 0.509039 |
| TCGA-R3-A69X-01A-22R-A30C-07 | 0.552139 |
| TCGA-UY-A9PB-01A-11R-A38B-07 | 0.79042 |
| TCGA-ZF-A9RD-01A-11R-A42T-07 | 0.7929 |
| TCGA-FD-A5BV-01A-11R-A26T-07 | 0.264705 |
| TCGA-XF-AAN2-01A-11R-A42T-07 | 0.648265 |
| TCGA-K4-A54R-01A-11R-A26T-07 | 0.713568 |
| TCGA-G2-A2EO-01A-11R-A180-07 | 0.662524 |
| TCGA-XF-A8HB-01A-11R-A36F-07 | 0.267618 |
| TCGA-XF-AAMH-01A-11R-A42T-07 | 0.276431 |
| TCGA-KQ-A41Q-01A-11R-A33J-07 | 0.250983 |
| TCGA-CF-A9FM-01A-11R-A38B-07 | 0.412791 |
| TCGA-XF-A9SV-01A-21R-A42T-07 | 0.301209 |
| TCGA-BT-A3PJ-01A-21R-A220-07 | 0.59652 |
| TCGA-CF-A3MF-01A-12R-A21D-07 | 0.219514 |
| TCGA-GD-A3OQ-01A-32R-A220-07 | 0.387337 |
| TCGA-XF-AAMR-01A-31R-A42T-07 | 0.444879 |
| TCGA-BT-A42C-01A-11R-A23N-07 | 0.231923 |
| TCGA-DK-A1AC-01A-11R-A13Y-07 | 0.721279 |
| TCGA-FD-A5C1-01A-11R-A28M-07 | 0.801498 |
| TCGA-BT-A3PH-01A-11R-A220-07 | 0.250338 |
| TCGA-FJ-A3ZF-01A-11R-A23N-07 | 0.089132 |
| TCGA-FD-A3SM-01A-11R-A22U-07 | 0.374616 |
| TCGA-FD-A3B7-01A-31R-A20F-07 | 0.875508 |
| TCGA-UY-A9PH-01A-11R-A38B-07 | 0.757672 |
| TCGA-BT-A42E-01A-11R-A23W-07 | 0.775593 |
| TCGA-XF-A9T4-01A-11R-A39I-07 | 0.63367 |
| TCGA-4Z-AA83-01A-11R-A39I-07 | 0.484854 |
| TCGA-FD-A3SQ-01A-21R-A22U-07 | 0.550338 |
| TCGA-FD-A3SO-01A-11R-A22U-07 | 0.516825 |
| TCGA-K4-AAQO-01A-11R-A38B-07 | 0.494891 |
| TCGA-FD-A6TC-01A-21R-A33J-07 | 0.35535 |
| TCGA-DK-A6B0-01A-11R-A31N-07 | 0.231985 |
| TCGA-GV-A3QG-01A-11R-A220-07 | 0.837181 |
| TCGA-4Z-AA86-01A-11R-A39I-07 | 0.770401 |
| TCGA-XF-A8HI-01A-11R-A38B-07 | 0.335938 |
| TCGA-4Z-AA80-01A-11R-A39I-07 | 0.322822 |
| TCGA-BL-A13I-01A-11R-A277-07 | 0.715563 |
| TCGA-E7-A7XN-01A-11R-A352-07 | 0.855866 |
| TCGA-C4-A0F6-01A-11R-A10U-07 | 0.372275 |
| TCGA-FD-A3B3-01A-12R-A206-07 | 0.783909 |
| TCGA-FD-A3B5-01A-11R-A20F-07 | 0.30212 |
| TCGA-ZF-A9R1-01A-11R-A39I-07 | 0.288709 |
| TCGA-ZF-AA4X-01A-11R-A38B-07 | 0.234328 |
| TCGA-UY-A9PE-01A-11R-A38B-07 | 0.301211 |
| TCGA-BT-A20N-01A-11R-A14Y-07 | 0.274156 |
| TCGA-GU-AATP-01A-11R-A39I-07 | 0.469759 |
| TCGA-XF-A9ST-01A-11R-A42T-07 | 0.183434 |
| TCGA-E5-A4TZ-01A-11R-A31N-07 | 0.259108 |
| TCGA-2F-A9KO-01A-11R-A38B-07 | 0.673907 |
| TCGA-YF-AA3L-01A-11R-A38B-07 | 0.356494 |
| TCGA-DK-A6AV-01A-12R-A30C-07 | 0.446014 |
| TCGA-K4-A3WV-01A-11R-A22U-07 | 0.333069 |
| TCGA-ZF-AA51-01A-21R-A39I-07 | 0.675161 |
| TCGA-CF-A1HS-01A-11R-A13Y-07 | 0.510558 |
| TCGA-ZF-A9R9-01A-11R-A38B-07 | 0.506049 |
| TCGA-GV-A3QI-01A-11R-A220-07 | 0.11951 |
| TCGA-UY-A8OB-01A-12R-A42T-07 | 0.561117 |
| TCGA-DK-A3X1-01A-12R-A22U-07 | 0.390451 |
| TCGA-BT-A3PK-01A-21R-A220-07 | 0.660023 |
| TCGA-DK-A6B6-01A-11R-A30C-07 | 0.590256 |
| TCGA-HQ-A2OF-01A-11R-A26T-07 | 0.163595 |
| TCGA-BT-A20T-01A-11R-A14Y-07 | 0.54698 |
| TCGA-BL-A13J-01A-11R-A277-07 | 0.344546 |
| TCGA-GU-A764-01A-11R-A352-07 | 0.681392 |
| TCGA-ZF-AA56-01A-31R-A39I-07 | 0.601895 |
| TCGA-XF-AAN5-01A-11R-A42T-07 | 0.7008 |
| TCGA-GC-A6I3-01A-11R-A31N-07 | 0.681612 |
| TCGA-G2-AA3D-01A-11R-A39I-07 | 0.287853 |
| TCGA-E7-A5KF-01A-11R-A28M-07 | 0.218484 |
| TCGA-GU-A762-01A-11R-A33J-07 | 0.881326 |
| TCGA-2F-A9KW-01A-11R-A38B-07 | 0.435344 |
| TCGA-DK-A2I2-01A-11R-A180-07 | 0.634968 |
| TCGA-FD-A5BY-01A-31R-A28M-07 | 0.451662 |
| TCGA-DK-AA6L-01A-11R-A39I-07 | 0.446931 |
| TCGA-FD-A3B4-01A-12R-A206-07 | 0.668397 |
| TCGA-XF-A9T6-01A-11R-A42T-07 | 0.466568 |
| TCGA-E7-A8O8-01A-11R-A36F-07 | 0.312587 |
| TCGA-FD-A3SS-01A-12R-A22U-07 | 0.222209 |
| TCGA-CU-A72E-01A-12R-A33J-07 | 0.390152 |
| TCGA-G2-A2EK-01A-22R-A18C-07 | 0.394409 |
| TCGA-XF-AAN7-01A-11R-A42T-07 | 0.452122 |
| TCGA-DK-A2I6-01A-12R-A18C-07 | 0.407319 |
| TCGA-S5-A6DX-01A-11R-A31N-07 | 0.73964 |
| TCGA-FD-A5BT-01A-11R-A26T-07 | 0.857809 |
| TCGA-DK-A1AA-01A-11R-A13Y-07 | 0.302241 |
| TCGA-FD-A6TD-01A-51R-A33J-07 | 0.723573 |
| TCGA-ZF-A9R3-01A-11R-A38B-07 | 0.490193 |
| TCGA-CU-A5W6-01A-11R-A28M-07 | 0.344041 |
| TCGA-XF-A9SX-01A-21R-A39I-07 | 0.822474 |
| TCGA-XF-AAN0-01A-11R-A42T-07 | 0.55331 |
| TCGA-FT-A3EE-01A-11R-A206-07 | 0.421 |
| TCGA-DK-AA75-01A-11R-A39I-07 | 0.166914 |
| TCGA-YC-A89H-01A-11R-A36F-07 | 0.410433 |
| TCGA-E5-A2PC-01A-11R-A206-07 | 0.484522 |
| TCGA-BT-A2LA-01A-11R-A18C-07 | 0.06037 |
| TCGA-FD-A6TA-01A-12R-A33J-07 | 0.622639 |
| TCGA-FD-A43X-01A-11R-A23W-07 | 0.168504 |
| TCGA-4Z-AA82-01A-11R-A39I-07 | 0.50787 |
| TCGA-GC-A3BM-01A-11R-A22U-07 | 0.359316 |
| TCGA-G2-A2EF-01A-12R-A18C-07 | 0.727411 |
| TCGA-G2-A2ES-01A-11R-A180-07 | 0.667661 |
| TCGA-XF-A8HG-01A-11R-A36F-07 | 0.250765 |
| TCGA-ZF-AA4V-01A-11R-A38B-07 | 0.656066 |
| TCGA-GV-A3QH-01A-11R-A220-07 | 0.138792 |
| TCGA-G2-A2EL-01A-12R-A18C-07 | 0.092643 |
| TCGA-K4-A83P-01A-11R-A352-07 | 0.821818 |
| TCGA-FD-A62S-01A-11R-A30C-07 | 0.672644 |
| TCGA-MV-A51V-01A-11R-A26T-07 | 0.149643 |
| TCGA-CF-A5UA-01A-11R-A28M-07 | 0.280566 |
| TCGA-4Z-AA7Q-01A-11R-A39I-07 | 0.740749 |
| TCGA-DK-A2I4-01A-11R-A21D-07 | 0.914186 |
| TCGA-BT-A20Q-01A-11R-A14Y-07 | 0.65584 |
| TCGA-BT-A42F-01A-11R-A23W-07 | 0.763778 |
| TCGA-E7-A85H-01A-11R-A352-07 | 0.545247 |
| TCGA-XF-AAME-01A-12R-A42T-07 | 0.754707 |
| TCGA-DK-AA6W-01A-12R-A39I-07 | 0.436323 |
| TCGA-FD-A43Y-01A-21R-A26T-07 | 0.557483 |
| TCGA-E7-A678-01A-11R-A30C-07 | 0.333681 |
| TCGA-G2-A2EC-01A-11R-A180-07 | 0.581519 |
| TCGA-FD-A5BX-01A-11R-A26T-07 | 0.647863 |
| TCGA-4Z-AA7W-01A-11R-A39I-07 | 0.928784 |
| TCGA-KQ-A41S-01A-12R-A33J-07 | 0.371585 |
| TCGA-K4-A5RH-01A-11R-A30C-07 | 0.89275 |
| TCGA-GV-A3QF-01A-31R-A22U-07 | 0.119754 |
| TCGA-GU-AATQ-01A-11R-A39I-07 | 0.448364 |
| TCGA-XF-AAMG-01A-11R-A42T-07 | 0.350617 |
| TCGA-4Z-AA87-01A-11R-A39I-07 | 0.537111 |
| TCGA-DK-AA74-01A-11R-A39I-07 | 0.871323 |
| TCGA-ZF-AA54-01A-11R-A39I-07 | 0.714271 |
| TCGA-FD-A6TG-01A-11R-A32O-07 | 0.496118 |
| TCGA-E7-A97Q-01A-11R-A38B-07 | 0.338375 |
| TCGA-FD-A3SN-01A-12R-A22U-07 | 0.467487 |
| TCGA-XF-A9SM-01A-11R-A42T-07 | 0.879089 |
| TCGA-UY-A9PD-01A-11R-A38B-07 | 0.386137 |
| TCGA-FJ-A871-01A-11R-A352-07 | 0.338698 |
| TCGA-XF-A9T0-01A-11R-A39I-07 | 0.46225 |
| TCGA-YC-A9TC-01A-22R-A39I-07 | 0.35885 |
| TCGA-XF-A9SH-01A-11R-A39I-07 | 0.431675 |
| TCGA-4Z-AA7O-01A-31R-A39I-07 | 0.506312 |
| TCGA-FD-A62O-01A-11R-A30C-07 | 0.25371 |
| TCGA-DK-AA6X-01A-12R-A42T-07 | 0.572392 |
| TCGA-E7-A97P-01A-11R-A38B-07 | 0.785683 |
| TCGA-4Z-AA7M-01A-11R-A39I-07 | 0.267905 |
| TCGA-BT-A0YX-01A-11R-A10U-07 | 0.633647 |
| TCGA-FD-A3B8-01A-31R-A20F-07 | 0.887617 |
| TCGA-C4-A0F7-01A-11R-A084-07 | 0.362303 |
| TCGA-ZF-A9RM-01A-11R-A38B-07 | 0.179697 |
| TCGA-GC-A3RB-01A-12R-A220-07 | 0.404422 |
| TCGA-BT-A20P-01A-11R-A14Y-07 | 0.221068 |
| TCGA-E7-A7DU-01A-11R-A32O-07 | 0.268686 |
| TCGA-DK-A3IK-01A-32R-A21D-07 | 0.306185 |
| TCGA-GV-A6ZA-01A-12R-A33J-07 | 0.438067 |
| TCGA-FD-A6TF-01A-52R-A32O-07 | 0.466723 |
| TCGA-GU-A763-01A-11R-A32O-07 | 0.252756 |
| TCGA-DK-AA6M-01A-11R-A39I-07 | 0.719918 |
| TCGA-CF-A47X-01A-31R-A23W-07 | 0.19023 |
| TCGA-DK-A6B5-01A-11R-A31N-07 | 0.508893 |
| TCGA-E7-A6MD-01A-41R-A352-07 | 0.5567 |
| TCGA-FJ-A3ZE-01A-11R-A23N-07 | 0.144039 |
| TCGA-UY-A78K-01A-11R-A33J-07 | 0.720979 |
| TCGA-XF-AAMT-01A-11R-A42T-07 | 0.515916 |
| TCGA-2F-A9KR-01A-11R-A38B-07 | 0.306039 |
| TCGA-XF-AAMZ-01A-11R-A42T-07 | 0.461735 |
| TCGA-ZF-A9RF-01A-11R-A38B-07 | 0.72901 |
| TCGA-UY-A78N-01A-12R-A33J-07 | 0.219606 |
| TCGA-ZF-AA53-01A-11R-A39I-07 | 0.711983 |
| TCGA-CF-A3MI-01A-11R-A20F-07 | 0.352755 |
| TCGA-E7-A677-01A-11R-A30C-07 | 0.496124 |
| TCGA-BT-A20W-01A-21R-A14Y-07 | 0.554388 |
| TCGA-LC-A66R-01A-41R-A30C-07 | 0.719034 |
| TCGA-CF-A7I0-01A-22R-A352-07 | 0.299798 |
| TCGA-XF-AAN8-01A-11R-A42T-07 | 0.730491 |
| TCGA-4Z-AA7Y-01A-11R-A39I-07 | 0.221948 |
| TCGA-ZF-A9R0-01A-11R-A38B-07 | 0.421499 |
| TCGA-ZF-A9R4-01A-11R-A38B-07 | 0.450147 |
| TCGA-GD-A6C6-01A-21R-A31N-07 | 0.406241 |
| TCGA-4Z-AA81-01A-11R-A39I-07 | 0.653157 |
| TCGA-ZF-A9R2-01A-11R-A39I-07 | 0.183934 |
| TCGA-E7-A4IJ-01A-31R-A26T-07 | 0.457761 |
| TCGA-FD-A5BR-01A-11R-A26T-07 | 0.615753 |
| TCGA-K4-A3WU-01B-11R-A23N-07 | 0.554162 |
| TCGA-C4-A0F1-01A-11R-A034-07 | 0.498576 |
| TCGA-ZF-A9R7-01A-11R-A38B-07 | 0.78202 |
| TCGA-CF-A47T-01A-11R-A23W-07 | 0.134609 |
| TCGA-GC-A6I1-01A-12R-A31N-07 | 0.83156 |
| TCGA-FD-A6TB-01A-12R-A33J-07 | 0.72261 |
| TCGA-E7-A6ME-01A-22R-A32O-07 | 0.357866 |
| TCGA-GU-A42P-01A-11R-A23W-07 | 0.113113 |
| TCGA-HQ-A2OE-01A-11R-A206-07 | 0.38414 |
| TCGA-K4-A5RI-01A-11R-A28M-07 | 0.457532 |
| TCGA-K4-A6MB-01A-11R-A31N-07 | 0.326988 |
| TCGA-XF-A9SJ-01A-11R-A39I-07 | 0.705275 |
| TCGA-XF-AAML-01A-11R-A42T-07 | 0.440586 |
| TCGA-FD-A5C0-01A-11R-A28M-07 | 0.362738 |
| TCGA-DK-A3IL-01A-11R-A20F-07 | 0.272258 |
| TCGA-XF-A8HC-01A-11R-A36F-07 | 0.097155 |
| TCGA-K4-A3WS-01A-11R-A22U-07 | 0.59169 |
| TCGA-E7-A4XJ-01A-11R-A26T-07 | 0.224151 |
| TCGA-ZF-AA5H-01A-11R-A39I-07 | 0.707836 |
| TCGA-FD-A3SP-01A-31R-A22U-07 | 0.771228 |
| TCGA-UY-A78O-01A-12R-A33J-07 | 0.355362 |
| TCGA-BT-A20J-01A-11R-A14Y-07 | 0.71019 |
| TCGA-ZF-AA4U-01A-11R-A38B-07 | 0.269543 |
| TCGA-UY-A78P-01A-12R-A36F-07 | 0.762465 |
| TCGA-CU-A3KJ-01A-11R-A21D-07 | 0.455722 |
| TCGA-FD-A43S-01A-21R-A23W-07 | 0.75141 |
| TCGA-CF-A9FF-01A-11R-A38B-07 | 0.410868 |
| TCGA-ZF-A9RN-01A-11R-A42T-07 | 0.685029 |
| TCGA-XF-A8HD-01A-11R-A36F-07 | 0.699321 |
| TCGA-DK-A6B1-01A-12R-A30C-07 | 0.244576 |
| TCGA-CU-A3YL-01A-11R-A22U-07 | 0.324612 |
| TCGA-CF-A9FH-01A-11R-A38B-07 | 0.245171 |
| TCGA-UY-A8OD-01A-11R-A36F-07 | 0.52608 |
| TCGA-E7-A8O7-01A-11R-A36F-07 | 0.464354 |
| TCGA-UY-A78L-01A-12R-A33J-07 | 0.464101 |
| TCGA-FD-A3SL-01A-21R-A22U-07 | 0.480282 |
| TCGA-K4-A4AB-01B-12R-A28M-07 | 0.4725 |
| TCGA-LT-A5Z6-01A-11R-A28M-07 | 0.400113 |
| TCGA-XF-A9T8-01A-11R-A39I-07 | 0.791103 |
| TCGA-XF-A9SP-01A-11R-A39I-07 | 0.475536 |
| TCGA-DK-A6B2-01A-11R-A30C-07 | 0.626463 |
| TCGA-BT-A20X-01A-11R-A16R-07 | 0.530679 |
| TCGA-CF-A8HY-01A-11R-A36F-07 | 0.276956 |
| TCGA-ZF-A9RE-01A-11R-A38B-07 | 0.34893 |
| TCGA-G2-AA3B-01A-11R-A39I-07 | 0.408749 |
| TCGA-CF-A3MG-01A-11R-A20F-07 | 0.098112 |
| TCGA-GV-A3JZ-01A-11R-A21D-07 | 0.434609 |
| TCGA-E7-A519-01A-11R-A26T-07 | 0.555987 |
| TCGA-GU-A767-01A-11R-A32O-07 | 0.29018 |
| TCGA-XF-A9SI-01A-11R-A39I-07 | 0.89161 |
| TCGA-5N-A9KM-01A-11R-A42T-07 | 0.496344 |
| TCGA-2F-A9KQ-01A-11R-A38B-07 | 0.199193 |
| TCGA-FD-A43U-01A-11R-A23W-07 | 0.722426 |
| TCGA-ZF-AA58-01A-12R-A42T-07 | 0.907073 |
| TCGA-CF-A1HR-01A-11R-A13Y-07 | 0.428379 |
| TCGA-FD-A62P-01A-32R-A30C-07 | 0.62644 |
| TCGA-E5-A4U1-01A-11R-A31N-07 | 0.180322 |
| TCGA-2F-A9KT-01A-11R-A38B-07 | 0.248659 |
| TCGA-UY-A78M-01A-21R-A352-07 | 0.3009 |
| TCGA-FD-A6TK-01A-42R-A33J-07 | 0.769689 |
| TCGA-GV-A40E-01A-12R-A23N-07 | 0.579085 |
| TCGA-FD-A3N6-01A-11R-A21D-07 | 0.52358 |
| TCGA-GU-A42Q-01A-11R-A23W-07 | 0.230576 |
| TCGA-BT-A0S7-01A-11R-A10U-07 | 0.374443 |
| TCGA-DK-A3IT-01A-31R-A20F-07 | 0.407841 |
| TCGA-ZF-A9RC-01A-11R-A38B-07 | 0.261199 |
| TCGA-FD-A6TE-01A-12R-A33J-07 | 0.39004 |
| TCGA-GU-A42R-01A-11R-A23N-07 | 0.097748 |
| TCGA-XF-A9T3-01A-11R-A42T-07 | 0.575641 |
| TCGA-DK-AA6P-01A-11R-A39I-07 | 0.235536 |
| TCGA-FD-A6TI-01A-11R-A32O-07 | 0.352491 |
| TCGA-FD-A3SR-01A-11R-A22U-07 | 0.579537 |
| TCGA-G2-A3IE-01A-11R-A20F-07 | 0.351909 |
| TCGA-G2-A2EJ-01A-11R-A180-07 | 0.603137 |
| TCGA-CF-A3MH-01A-11R-A20F-07 | 0.235131 |
| TCGA-DK-AA6S-01A-21R-A39I-07 | 0.663554 |
| TCGA-LT-A8JT-01A-11R-A36F-07 | 0.358648 |
| TCGA-UY-A9PF-01A-11R-A38B-07 | 0.46795 |
| TCGA-GC-A4ZW-01A-11R-A26T-07 | 0.321046 |
| TCGA-DK-AA77-01A-11R-A39I-07 | 0.450716 |
| TCGA-FD-A3N5-01A-11R-A21D-07 | 0.43086 |
| TCGA-CF-A27C-01A-11R-A16R-07 | 0.193057 |
| TCGA-CU-A3QU-01A-11R-A22U-07 | 0.252605 |
| TCGA-KQ-A41P-01A-12R-A33J-07 | 0.462811 |
| TCGA-GV-A3JW-01A-11R-A20F-07 | 0.044838 |
| TCGA-XF-AAN4-01A-11R-A42T-07 | 0.737948 |
| TCGA-SY-A9G0-01A-12R-A38B-07 | 0.514125 |
| TCGA-4Z-AA89-01A-11R-A39I-07 | 0.31841 |
| TCGA-CF-A47Y-01A-11R-A23W-07 | 0.178271 |
| TCGA-DK-A3WX-01A-22R-A22U-07 | 0.624253 |
| TCGA-FT-A61P-01A-11R-A30C-07 | 0.634861 |
| TCGA-E7-A5KE-01A-11R-A28M-07 | 0.172865 |
| TCGA-E7-A6MF-01A-12R-A32O-07 | 0.333392 |
| TCGA-GV-A3JV-01A-11R-A220-07 | 0.592937 |
| TCGA-K4-A5RJ-01A-11R-A28M-07 | 0.907354 |
| TCGA-DK-A1AD-01A-11R-A13Y-07 | 0.380401 |
| TCGA-XF-AAN3-01A-11R-A42T-07 | 0.553523 |
| TCGA-BT-A20V-01A-11R-A14Y-07 | 0.577242 |
| TCGA-DK-A3IV-01A-22R-A21D-07 | 0.668447 |
| TCGA-BT-A2LB-01A-11R-A18C-07 | 0.548978 |
| TCGA-YC-A8S6-01A-31R-A38B-07 | 0.651165 |
| TCGA-CF-A8HX-01A-11R-A36F-07 | 0.276423 |
| TCGA-GV-A40G-01A-11R-A23N-07 | 0.139122 |
| TCGA-GU-AATO-01A-11R-A39I-07 | 0.526268 |
| TCGA-HQ-A5ND-01A-11R-A26T-07 | 0.370231 |
| TCGA-5N-A9KI-01A-31R-A42T-07 | 0.459332 |
| TCGA-BT-A20R-01A-12R-A16R-07 | 0.415058 |
| TCGA-KQ-A41O-01A-12R-A352-07 | 0 |
| TCGA-XF-A9T5-01A-11R-A42T-07 | 0.824316 |
| TCGA-DK-A2HX-01A-12R-A18C-07 | 0.464515 |
| TCGA-XF-AAMW-01A-11R-A42T-07 | 0.604072 |
| TCGA-GC-A3I6-01A-11R-A20F-07 | 0.58117 |
| TCGA-XF-A9SW-01A-11R-A42T-07 | 0.499487 |
| TCGA-CU-A0YR-01A-12R-A10U-07 | 0.603003 |
| TCGA-DK-A3IM-01A-11R-A20F-07 | 0.275974 |
| TCGA-GC-A3RD-01A-12R-A22U-07 | 0.259214 |
| TCGA-HQ-A5NE-01A-12R-A28M-07 | 0.469225 |
| TCGA-G2-A3IB-01A-11R-A20F-07 | 0.398614 |
| TCGA-FD-A62N-01A-11R-A30C-07 | 1 |
| TCGA-DK-AA6U-01A-11R-A39I-07 | 0.300482 |
| TCGA-BT-A2LD-01A-12R-A20F-07 | 0.480283 |
| TCGA-BT-A20U-01A-11R-A14Y-07 | 0.4893 |
| TCGA-GV-A3JX-01A-11R-A20F-07 | 0.496265 |
| TCGA-DK-A1AB-01A-11R-A13Y-07 | 0.637348 |
| TCGA-SY-A9G5-01A-11R-A38B-07 | 0.769266 |
| TCGA-PQ-A6FN-01A-11R-A31N-07 | 0.494513 |
| TCGA-XF-A9SU-01A-31R-A39I-07 | 0.51665 |
| TCGA-BL-A5ZZ-01A-31R-A30C-07 | 0.587687 |
| TCGA-S5-AA26-01A-11R-A38B-07 | 0.213283 |
| TCGA-C4-A0EZ-01A-21R-A24X-07 | 0.166929 |
| TCGA-FD-A6TH-01A-11R-A32O-07 | 0.412993 |
| TCGA-GU-A766-01A-11R-A32O-07 | 0.990318 |
| TCGA-CU-A0YN-01A-21R-A10U-07 | 0.549884 |
| TCGA-FD-A5BS-01A-21R-A26T-07 | 0.999499 |
| TCGA-K4-A6FZ-01A-11R-A31N-07 | 0.58448 |
| TCGA-DK-A3IS-01A-21R-A21D-07 | 0.297994 |
| TCGA-DK-A1A7-01A-11R-A13Y-07 | 0.332181 |
| TCGA-2F-A9KP-01A-11R-A38B-07 | 0.27828 |
| TCGA-BL-A0C8-01A-11R-A277-07 | 0.195408 |
| TCGA-FD-A5BU-01A-31R-A26T-07 | 0.63678 |
| TCGA-XF-AAMJ-01A-11R-A42T-07 | 0.522163 |
| TCGA-4Z-AA7S-01A-11R-A39I-07 | 0.202182 |
| TCGA-DK-AA6Q-01A-11R-A39I-07 | 0.671715 |
| TCGA-H4-A2HO-01A-11R-A180-07 | 0.472308 |
| TCGA-DK-A3X2-01A-11R-A22U-07 | 0.217045 |
| TCGA-DK-A1AF-01A-11R-A13Y-07 | 0.616423 |
| TCGA-DK-AA6R-01A-11R-A42T-07 | 0.49774 |
| TCGA-GC-A3WC-01A-31R-A22U-07 | 0.748659 |
| TCGA-FD-A5BZ-01A-11R-A28M-07 | 0.434691 |
| TCGA-ZF-AA4W-01A-12R-A38B-07 | 0.448524 |
| TCGA-KQ-A41R-01A-21R-A352-07 | 0.264327 |
| TCGA-G2-AA3F-01A-12R-A42T-07 | 0.303038 |
| TCGA-ZF-AA5N-01A-11R-A42T-07 | 0.42717 |
| TCGA-FD-A3SJ-01A-12R-A22U-07 | 0.331733 |
| TCGA-GD-A2C5-01A-12R-A180-07 | 0.312338 |
| TCGA-4Z-AA84-01A-11R-A39I-07 | 0.38407 |
| TCGA-XF-A8HH-01A-11R-A38B-07 | 0.426248 |
| TCGA-KQ-A41N-01A-11R-A33J-07 | 0.14975 |
| TCGA-ZF-AA5P-01A-11R-A39I-07 | 0.514226 |
| TCGA-XF-A9SL-01A-11R-A39I-07 | 0.663111 |
| TCGA-BT-A20O-01A-21R-A14Y-07 | 0.974947 |
| TCGA-DK-A1A5-01A-11R-A13Y-07 | 0.442115 |
| TCGA-ZF-AA4T-01A-11R-A38B-07 | 0.305229 |
| TCGA-DK-AA71-01A-31R-A39I-07 | 0.342962 |
| TCGA-GD-A3OP-01A-21R-A220-07 | 0.408633 |
| TCGA-FD-A43N-01A-11R-A23W-07 | 0.446326 |
| TCGA-FD-A3B6-01A-21R-A20F-07 | 0.70605 |
| TCGA-DK-A6AW-01A-11R-A30C-07 | 0.412817 |
| TCGA-GD-A76B-01A-11R-A32O-07 | 0.623696 |
| TCGA-DK-A3WY-01A-11R-A22U-07 | 0.95005 |
| TCGA-FD-A3NA-01A-11R-A21D-07 | 0.531284 |
| TCGA-XF-AAN1-01A-31R-A42T-07 | 0.302804 |
| TCGA-XF-AAMQ-01A-11R-A42T-07 | 0.668097 |
| TCGA-DK-A1A6-01A-11R-A13Y-07 | 0.591414 |
| TCGA-G2-A3VY-01A-11R-A22U-07 | 0.073777 |
| TCGA-UY-A8OC-01A-11R-A36F-07 | 0.454863 |
| TCGA-E7-A3X6-01A-12R-A22U-07 | 0.640592 |
| TCGA-CF-A47S-01A-11R-A23W-07 | 0.398612 |
| TCGA-XF-AAMY-01A-11R-A42T-07 | 0.315129 |
| TCGA-FJ-A3Z7-01A-12R-A23N-07 | 0.47037 |
| TCGA-XF-A9SZ-01A-11R-A39I-07 | 0.486134 |
| TCGA-DK-AA6T-01A-11R-A39I-07 | 0.713059 |
| TCGA-XF-A9SY-01A-21R-A42T-07 | 0.833892 |
| TCGA-CF-A9FL-01A-11R-A38B-07 | 0.296014 |
| TCGA-4Z-AA7R-01A-11R-A39I-07 | 0.220825 |
| TCGA-XF-A8HE-01A-11R-A36F-07 | 0.719513 |
| TCGA-DK-A1AE-01A-11R-A13Y-07 | 0.451508 |
| TCGA-XF-A9T2-01A-11R-A42T-07 | 0.310245 |
| TCGA-BL-A3JM-01A-12R-A21D-07 | 0.369826 |
| TCGA-XF-AAMX-01A-11R-A42T-07 | 0.368729 |
| TCGA-H4-A2HQ-01A-11R-A180-07 | 0.274169 |
| TCGA-PQ-A6FI-01A-11R-A31N-07 | 0.500191 |
| TCGA-G2-AA3C-01A-21R-A39I-07 | 0.643744 |
| TCGA-CF-A5U8-01A-11R-A28M-07 | 0.16604 |
| TCGA-C4-A0F0-01A-12R-A10U-07 | 0.560045 |
| TCGA-XF-A8HF-01A-11R-A36F-07 | 0.474505 |
| TCGA-DK-A1AG-01A-11R-A13Y-07 | 0.341757 |
| TCGA-DK-A3IQ-01A-31R-A32Y-07 | 0.354678 |
| TCGA-FJ-A3Z9-01A-11R-A26T-07 | 0.187959 |
| TCGA-DK-A3IU-01A-11R-A20F-07 | 0.896709 |
| TCGA-GD-A3OS-01A-12R-A220-07 | 0.392649 |
| TCGA-GC-A3YS-01A-11R-A23N-07 | 0.524602 |
| TCGA-GC-A3OO-01A-11R-A22U-07 | 0.377094 |
| TCGA-DK-A3IN-01A-11R-A20F-07 | 0.567927 |
| TCGA-DK-A3WW-01A-22R-A23N-07 | 0.828604 |
| TCGA-DK-A1A3-01A-11R-A13Y-07 | 0.548535 |
| TCGA-FD-A43P-01A-31R-A23W-07 | 0.738474 |
| TCGA-E7-A3Y1-01A-11R-A22U-07 | 0.285961 |
| TCGA-XF-A9SK-01A-11R-A42T-07 | 0.628877 |
